# Supplementary material for: Non-invasive vocal biomarker is associated with pulmonary hypertension
Source: PLoS One. 2020 Apr 16;15(4):e0231441. doi: 10.1371/journal.pone.0231441 (PMC7162478; doi:10.1371/journal.pone.0231441)
Supplement: Attachment — Submitted filename: Voice_PulmonaryHTN_Responsetoreviewers.docx [file pone.0231441.s002.docx]

Vincenzo Lionetti, M.D., PhD
Academic Editor
PLOS ONE

**Manuscript. Ref. No.:**  PONE-D-19-27216 **Title:** Non-Invasive Vocal Biomarker is Associated with Pulmonary Hypertension

Dear Dr. Lionetti,

Thank you very much for the thoughtful review of our manuscript. We hope that we are able to adequately address the reviewer’s comments and that you will consider the paper acceptable for publication.

Please find our responses to the reviewer’s comments below:

**Journal Requirements:**

When submitting your revision, we need you to address these additional requirements.
Please ensure that your manuscript meets PLOS ONE's style requirements, including those for file naming. The PLOS ONE style templates can be found at
<http://www.journals.plos.org/plosone/s/file?id=wjVg/PLOSOne_formatting_sample_main_body.pdf> and <http://www.journals.plos.org/plosone/s/file?id=ba62/PLOSOne_formatting_sample_title_authors_affiliations.pdf>

We thank the editor for the comment. We have revised the manuscript accordingly.

Thank you for stating the following in the Financial Disclosure section:

Dr. Elad Maor serves as a consultant for Beyond Verbal Communications.  The remaining authors have nothing to disclose

We note that one or more of the authors are employed by a commercial company: Beyond Verbal Communications

1. Please provide an amended Funding Statement declaring this commercial affiliation, as well as a statement regarding the Role of Funders in your study. If the funding organization did not play a role in the study design, data collection and analysis, decision to publish, or preparation of the manuscript and only provided financial support in the form of authors' salaries and/or research materials, please review your statements relating to the author contributions, and ensure you have specifically and accurately indicated the role(s) that these authors had in your study. You can update author roles in the Author Contributions section of the online submission form.

Please also include the following statement within your amended Funding Statement.

“The funder provided support in the form of salaries for authors [insert relevant initials], but did not have any additional role in the study design, data collection and analysis, decision to publish, or preparation of the manuscript. The specific roles of these authors are articulated in the ‘author contributions’ section.”

If your commercial affiliation did play a role in your study, please state and explain this role within your updated Funding Statement.

We thank the editor for the comment. Please note that Dr. Elad Maor serves as a consultant for Beyond Verbal Communications, but does not receive any salary from the company. Beyond Verbal Communications provided some of the funding for the study, but did not play a role in the study design, data collection and analysis, decision to publish, or preparation of the manuscript, and only provided financial support in the form of study funding. As such we have modified our Funding Statement as follows:

“This study was in part funded by Beyond Verbal Communications. The funders had no role in study design, data collection and analysis, decision to publish, or preparation of the manuscript. Dr. Elad Maor serves as a consultant for Beyond Verbal Communications. This author received no specific funding for this work. The remaining authors have nothing to disclose.”

2. Please also provide an updated Competing Interests Statement declaring this commercial affiliation along with any other relevant declarations relating to employment, consultancy, patents, products in development, or marketed products, etc.

Within your Competing Interests Statement, please confirm that this commercial affiliation does not alter your adherence to all PLOS ONE policies on sharing data and materials by including the following statement: "This does not alter our adherence to  PLOS ONE policies on sharing data and materials.” (as detailed online in our guide for authors <http://journals.plos.org/plosone/s/competing-interests>) . If this adherence statement is not accurate and there are restrictions on sharing of data and/or materials, please state these. Please note that we cannot proceed with consideration of your article until this information has been declared.

Please include both an updated Funding Statement and Competing Interests Statement in your cover letter. We will change the online submission form on your behalf.

Please know it is PLOS ONE policy for corresponding authors to declare, on behalf of all authors, all potential competing interests for the purposes of transparency. PLOS defines a competing interest as anything that interferes with, or could reasonably be perceived as interfering with, the full and objective presentation, peer review, editorial decision-making, or publication of research or non-research articles submitted to one of the journals. Competing interests can be financial or non-financial, professional, or personal. Competing interests can arise in relationship to an organization or another person. Please follow this link to our website for more details on competing interests: <http://journals.plos.org/plosone/s/competing-interests>

We thank the editor for the comment. Please note that Dr. Elad Maor serves as a consultant for Beyond Verbal Communications, but does not receive any salary from the company. Beyond Verbal Communications provided some of the funding for the study, but did not play a role in the study design, data collection and analysis, decision to publish, or preparation of the manuscript, and only provided financial support in the form of study funding. As such we have modified our Competing Interests Statement as follows:

“Dr. Elad Maor serves as a consultant for Beyond Verbal Communications. This author receives no salary for this work. The remaining authors have nothing to disclose. This study was in part funded by Beyond Verbal Communications, who had no role in study design, data collection and analysis, decision to publish, or preparation of the manuscript.”

 3. In your Data Availability statement, you have not specified where the minimal data set underlying the results described in your manuscript can be found. PLOS defines a study's minimal data set as the underlying data used to reach the conclusions drawn in the manuscript and any additional data required to replicate the reported study findings in their entirety. All PLOS journals require that the minimal data set be made fully available. For more information about our data policy, please see <http://journals.plos.org/plosone/s/data-availability>.

Upon re-submitting your revised manuscript, please upload your study’s minimal underlying data set as either Supporting Information files or to a stable, public repository and include the relevant URLs, DOIs, or accession numbers within your revised cover letter. For a list of acceptable repositories, please see <http://journals.plos.org/plosone/s/data-availability#loc-recommended-repositories>. Any potentially identifying patient information must be fully anonymized.

Important: If there are ethical or legal restrictions to sharing your data publicly, please explain these restrictions in detail. Please see our guidelines for more information on what we consider unacceptable restrictions to publicly sharing data: <http://journals.plos.org/plosone/s/data-availability#loc-unacceptable-data-access-restrictions>. Note that it is not acceptable for the authors to be the sole named individuals responsible for ensuring data access.

We will update your Data Availability statement to reflect the information you provide in your cover letter.

We thank the editor for this comment. Please note we have submitted our minimal dataset as a Supporting Information file, entitled S1 Dataset.

[Note: HTML markup is below. Please do not edit.]

Reviewers' comments:

Reviewer's Responses to Questions

**Comments to the Author**

1. Is the manuscript technically sound, and do the data support the conclusions?

The manuscript must describe a technically sound piece of scientific research with data that supports the conclusions. Experiments must have been conducted rigorously, with appropriate controls, replication, and sample sizes. The conclusions must be drawn appropriately based on the data presented.

Reviewer #1: Partly

2. Has the statistical analysis been performed appropriately and rigorously?

Reviewer #1: No

3. Have the authors made all data underlying the findings in their manuscript fully available?

The [PLOS Data policy](http://www.plosone.org/static/policies.action#sharing) requires authors to make all data underlying the findings described in their manuscript fully available without restriction, with rare exception (please refer to the Data Availability Statement in the manuscript PDF file). The data should be provided as part of the manuscript or its supporting information, or deposited to a public repository. For example, in addition to summary statistics, the data points behind means, medians and variance measures should be available. If there are restrictions on publicly sharing data—e.g. participant privacy or use of data from a third party—those must be specified.

Reviewer #1: No

4. Is the manuscript presented in an intelligible fashion and written in standard English?

PLOS ONE does not copyedit accepted manuscripts, so the language in submitted articles must be clear, correct, and unambiguous. Any typographical or grammatical errors should be corrected at revision, so please note any specific errors here.

Reviewer #1: Yes

5. Review Comments to the Author

Please use the space provided to explain your answers to the questions above. You may also include additional comments for the author, including concerns about dual publication, research ethics, or publication ethics. (Please upload your review as an attachment if it exceeds 20,000 characters)

Reviewer #1: The authors propose to use a voice biomarker to examine an association between levels of the voice biomarker and HF.

The voice biomarker is an unbounded scalar. The authors mention using a univariate analysis and also a multivariate analysis (both using repeated measures) on the data. However, it is unclear which analytic method is used. Is it some form of linear regression (since the outcome voice biomarker is a scalar)? Is it some form of logistic regression (since the authors report odds ratios)? Is it some form of generalized estimating equations (since the authors mention using repeated measures)? It is unclear which method was used for the univariate & multivariate analyses. The authors need to state the method used before a reviewer can determine if it is appropriate or not.

We thank the reviewer for the comment. The primary outcome of interest in the current study was the dependent variable moderate or severely elevated pulmonary arterial pressure, defined as a pulmonary artery pressure ≥ 35 mmHg. As a secondary outcome, we also looked at elevated pulmonary vascular resistance, defined as ≥ 1.7 Wood Units. As such, the dependent variables in the current study were binary categorical variables, and so we used logistic regression to determine the odds of having a moderate or severely elevated pulmonary arterial pressure and the odds of having an elevated pulmonary vascular resistance with the vocal biomarker as the independent variable. We have included the following comments in our statistical analysis section of the materials and methods.

“Univariate logistic regression analyses were undertaken to determine the association between the voice biomarker, as the independent variable, and each of the following individually as categorical dependent variables: a high PAP, PVR, PWCP, RA pressure, and CI. Each association was examined in all patients and after stratifying by a high versus lower PCWP. The distinction between high and lower PCWP was chosen to distinguish PH that was “post-capillary” in etiology and therefore related to coexisting left HF versus that which was “pre-capillary” in etiology and therefore related to a primary vascular and/or lung pathology. Finally, multivariate logistic regression analyses with repeated measures were undertaken to determine the relationships between the voice biomarker, as the independent variable, and each of the following individually as categorical dependent variables: a high PAP, PVR, PWCP, RA pressure, and CI. Each association was examined in all patients and after stratifying by a high versus lower PCWP. Each analysis was adjusted for age, sex, hypertension, diabetes mellitus, and NYHA class as these factors are known to be associated with PH and/or HF and could therefore act as potential confounders.”

Additionally, the authors state that they used Student's t-test to analyze normally distributed, continuous data. However, from the boxplot for < 1.7 in Figure 2B, it appears that data is highly skewed and may not be normally distributed. Did the authors check the normality assumptions before running the student's t-tests? If so, that should be stated. If not, it should be done, and any transformations needed should be performed. If after transforming, the data is still not normally distributed, then a Mann-Whitney test should be used instead.

We thank the reviewer for the comment. We have added the following comment to the statistical analysis sub-section of the materials and methods:

“Normal distribution and equal variance were checked by the Shapro-Wills test, and Levene’s test respectively for each variable.”

For all figures/tables, the meaning of * should be stated in the table/figure description. Additionally, for tables, it should be stated what values are present in the 2nd column (mean plus/minus SD, or percent of total).

We thank the reviewer for the comments. We have amended the figure captions and tables accordingly.

 6. PLOS authors have the option to publish the peer review history of their article ([what does this mean?](https://journals.plos.org/plosone/s/editorial-and-peer-review-process#loc-peer-review-history)). If published, this will include your full peer review and any attached files.


If you choose “no”, your identity will remain anonymous but your review may still be made public.


**Do you want your identity to be public for this peer review?** For information about this choice, including consent withdrawal, please see our [Privacy Policy](https://www.plos.org/privacy-policy).

Reviewer #1: No
