# Supplementary material for: Non-invasive vocal biomarker is associated with pulmonary hypertension
Source: PLoS One. 2020 Apr 16;15(4):e0231441. doi: 10.1371/journal.pone.0231441 (PMC7162478; doi:10.1371/journal.pone.0231441)
Supplement: Attachment — Submitted filename: Voice_PulmonaryHTN_ResponsetoreviewersR1.docx [file pone.0231441.s003.docx]

Vincenzo Lionetti, M.D., PhD
Academic Editor
PLOS ONE

**Manuscript. Ref. No.:**  PONE-D-19-27216R1 **Title:** Non-Invasive Vocal Biomarker is Associated with Pulmonary Hypertension

Dear Dr. Lionetti,

Thank you very much for the thoughtful review of our revised manuscript. We hope that we are able to adequately address the reviewer’s comments and that you will consider the paper acceptable for publication.

Please find our responses to the reviewer’s comments below:

Reviewers' comments:

Reviewer's Responses to Questions

**Comments to the Author**

1. If the authors have adequately addressed your comments raised in a previous round of review and you feel that this manuscript is now acceptable for publication, you may indicate that here to bypass the “Comments to the Author” section, enter your conflict of interest statement in the “Confidential to Editor” section, and submit your "Accept" recommendation.

Reviewer #1: (No Response)
 

2. Is the manuscript technically sound, and do the data support the conclusions?

The manuscript must describe a technically sound piece of scientific research with data that supports the conclusions. Experiments must have been conducted rigorously, with appropriate controls, replication, and sample sizes. The conclusions must be drawn appropriately based on the data presented.

Reviewer #1: No

3. Has the statistical analysis been performed appropriately and rigorously?

Reviewer #1: No

4. Have the authors made all data underlying the findings in their manuscript fully available?

The [PLOS Data policy](http://www.plosone.org/static/policies.action#sharing) requires authors to make all data underlying the findings described in their manuscript fully available without restriction, with rare exception (please refer to the Data Availability Statement in the manuscript PDF file). The data should be provided as part of the manuscript or its supporting information, or deposited to a public repository. For example, in addition to summary statistics, the data points behind means, medians and variance measures should be available. If there are restrictions on publicly sharing data—e.g. participant privacy or use of data from a third party—those must be specified.

Reviewer #1: Yes

5. Is the manuscript presented in an intelligible fashion and written in standard English?

PLOS ONE does not copyedit accepted manuscripts, so the language in submitted articles must be clear, correct, and unambiguous. Any typographical or grammatical errors should be corrected at revision, so please note any specific errors here.

Reviewer #1: Yes

6. Review Comments to the Author

Reviewer #1: This reviewer thanks the authors for submitting the data as supplemental information as well as the clarification of methods used. Based on the data provided, it is not clear that the analysis performed is appropriate. The data provided contain information from at most three separate recordings. The authors do not mention how they deal with this repeated measures data in the analysis. This needs to be corrected and dealt with. It is highly suggested that the authors consult with a statistician before proceeding.

*We thank the reviewer for the comment. We have consulted with a statistician, who has been added to the manuscript as a co-author. We agree that our previous statistical methods may have erroneously inflated the power of our analyses, as we had undertaken the primary analyses for all separate individual voice recordings. We have changed the analysis now by calculating the mean voice biomarker value from the three separate recordings for each individual study participant, and have used this mean value in the subsequent analyses. We elected the purse the mean for two reasons: we found very good agreement between repeated recordings amongst our cohort (the intra-class correlation coefficient was 0.829 (95% CI 0.740 – 0.889) showing that our repeated voice biomarker values were stable and reliable and in turn correlated well with each other. Thus the mean provides a reasonable summary statistic for the multiple recordings. Secondly, in our previous study (in press) in which we showed an association between the same voice biomarker and HF outcomes including rehosptalization, we did not show any differences in the association between the voice biomarker and clinical outcomes when the voice biomarker used was derived from individuals recording their voices talking about positive, negative, or neutral experiences separately. Thus there did not seem to be a reasonable necessity on which to test the association between the voice biomarker derived under the three separate circumstances and the indices related to pulmonary hypertension in the current study. Nevertheless, we do present data in the results section of the manuscript showing the association between the voice biomarker derived whilst talking about a negative, positive and neutral experience and mean pulmonary artery pressure and pulmonary vascular resistance. We were able to do this in a univariable analyses but not multivariable analyses due to limitations of sample sizes. Please see below for the additions to the manuscript:*

After excluding poor quality recordings, values of the pre-specified voice biomarker were obtained from each high quality recording for each patient. In our recent study in which we showed that the same pre-specified voice biomarker was associated with increased mortality and re-hospitalization in patients with HF [28] (manuscript in press), we did not show any significant differences in the association between the voice biomarker and clinical outcomes when the voice biomarker used was derived from individuals recording their voices talking about positive, negative, or neutral experiences separately. Consequently, we elected to determine the agreement across the separate voice biomarker values for each individual participant by calculating the intra-class correlation coefficient with 95% confidence interval. We then calculated the mean voice biomarker value for each patient and used these numbers in our final analyses. The mean biomarker values were then compared between groups using Student’s t-test.

A pre-established vocal biomarker was determined based on each separate recording for each individual participant. The intra-class correlation co-efficient between the separate voice recording biomarker values for each individual participant was 0.829 (95% CI 0.740 – 0.889) implying very good agreement between values. Thus, the mean biomarker was calculated for each patient.

Table 2 shows the results of a univariable analysis evaluating the association between hemodynamic indices and the mean voice biomarker among all patients and after stratifying by high versus low PCWP. In all patients, the mean voice biomarker was significantly associated with a mean PAP ≥ 35 mmHg such that an increase in the mean biomarker by 1 unit was associated with an odds ratio (95% CI) of 1.92 (1.00 – 3.65, p=0.049). This association did not persist after stratifying by high or low PCWP. In a separate univariable analysis, the biomarker derived while describing a negative experience was significantly associated with a high mean PAP, odds ratio (95% CI) 1.91 (1.05 – 3.49, p=0.035) but not when the biomarker was derived while describing a neutral experience, odds ratio (95% CI) 1.31 (0.69 – 2.49, p=414) or positive experience, odds ratio (95% CI) 1.53 (0.85 – 2.76, p=016). The mean voice biomarker was not significantly associated with a PVR ≥ 1.7 Wood Units amongst all patients or in those after stratifying by high and low PCWP. Further, the voice biomarker was not significantly associated with a high PVR when it was derived in individuals describing a negative experience, odds ratio (95% CI) 1.54 (0.88 – 2.71, p=0.13), neutral experience, odds ratio (95% CI) 1.57 (0.76 – 3.23, p=0.223) or positive experience, odds ratio (95% CI) 1.44 (0.75 – 2.75, p=0.272). The voice biomarker was not significantly associated with any other hemodynamic index.

Student's t-tests are used. However, this is not the appropriate method for repeated measures data. Paired t-tests (when only 2 time points are present) or a 2-way repeated measures ANOVA are appropriate. IF the authors averaged the data at the three (or sometimes 2) time points, this should instead be stated, and justified as to why that is allowable/appropriate here. The text should also be modified if the values are averaged, as the voice characteristics section makes a point that each of the three recordings are distinct for a reason. If only one of the voice recordings was used for the analysis, the exact recording used should be stated instead.

*We thank the reviewer for the comment. We elected to calculate the mean value of the voice biomarker for each individual participant in the cohort. Please see above for details.*

It is noted that some patients do not have high quality recordings for all three time points. They instead only have 2 measurements. No mention is given to how this is handled in the analysis (are only 2 values averaged or is that patient removed from the analysis?). Additionally, some patients do not have a PVR value. No mention is provided as to how this is handled. Is the dataset for the primary outcome different from the dataset for the secondary outcome? If so, this should be stated, and mentioned how they differ. The alternate is to use the same (reduced) dataset for both the primary outcome and secondary outcome.

*We thank the reviewer for the comment. We have included the following statement to clarify our statistical methods:*

In cases in which a study participant had one or more voice recording samples excluded due to poor quality, the remaining high-quality samples were retained and used to determine the mean biomarker value for that patient.

The same dataset was used for all analyses.

There is no such method as a multivariate logistic regression with repeated measures. The use of this term should be corrected to the appropriate method actually used. Logistic regression does not account for repeated measures, and it is not possible for that specific method to take them into account. Other methods can. If those were used, that needs to be indicated.

*We thank the reviewer for the comment. We agree and thus have removed this statement from the manuscript. We have elected to undertake our analyses using the mean value of the voice biomarker for each patient, and then undertook univariable and multivariable logistic regression analyses using these values. See above for more details.*

From looking at the results, it appears that the (up to) 3 biomarker values were averaged, and a single model for the primary outcome and different model for the secondary outcome was created. If this is correct, this needs to be stated and HIGHLY justified as it is not frequently appropriate to average across different recordings when each means something different.

*We thank the reviewer for the comment. Please see above for more details. We elected to determine the mean value of the voice biomarker for each individual participant, because the mean value provided a reasonable and appropriate summary statistic for each patient given that the intra-class correlation coefficient that we calculated showed good agreement between values. This provided evidence that the separate voice recordings for each patient were stable and reliable, and therefore suitable to combine to obtain a mean value. Furthermore our previous study in this area did not show that voice biomarker values obtained under different circumstances (describing a neutral, positive or negative situation) were differently associated with heart failure clinical outcomes, and thus we did not intend to primarily test the different associations between the voice biomarker under different circumstances and the invasive indices measured in this study. Thus, we elected to use the mean values in our subsequent primary analyses, and consequently have changed our final results and figures accordingly. In addition, we assessed the association between the mean voice biomarker value and mean PAP and PVR separately in two different models. Mean PAP was our primary outcome of interest as it is the marker that facilitates the diagnosis of pulmonary hypertension itself and characterizes its severity. PVR is a useful adjunct measure when looking at pulmonary hypertension, but is not of primary importance when making the diagnosis or determining the severity of pulmonary hypertension. Further, these two measures are related to each other and we did not want to have them present in the same model as both markers could account for at least some of the same proportion of variability of the voice biomarker across different values.*

A mixed effects model or generalized estimating equation approach might be more appropriate here since there are repeated measurements. Alternately, the authors might consider doing an analysis separately on each time recording (i.e. an analysis for recording 1, an analysis for recording 2, and an analysis for recording 3). This is where consulting with a statistician will be beneficial as they can best guide based on the specific questions and data at hand.

*We thank the reviewer for the comment. Please see above for more details. We describe the results of the association between the mean value of the voice biomarker and mean PAP and PVR. We also show in our results section the findings of the association between the value of the voice marker derived when individuals are describing a negative experience, positive experience, and neutral experience and mean PAP and PVR in univariable analyse..*
